# *Pentaclethra macroloba*: A Review of the Biological, Pharmacological, Phytochemical, Cosmetic, Nutritional and Biofuel Potential of this Amazonian Plant

**DOI:** 10.3390/plants12061330

**Published:** 2023-03-15

**Authors:** Maria Louze Nobre Lamarão, Lindalva Maria de Meneses Costa Ferreira, Desireé Gyles Lynch, Luiz Roberto Barbosa Morais, José Otávio Carréra Silva-Júnior, Roseane Maria Ribeiro-Costa

**Affiliations:** 1 Laboratory of Pharmaceutical Nanotechnology, College of Pharmacy, Federal University of Pará, Belém 66075-110, Pará, Brazil; 2 School of Pharmacy, College of Health Sciences, School of Pharmacy, University of Technology, 237 Old Hope Road, Kingston 6, Jamaica; 3 Amazon Oil Industry, Levilândia, Ananindeua 67015-760, Pará, Brazil; 4 Laboratory of Pharmaceutical and Cosmetic R&D, College of Pharmacy, Federal University of Pará, Belém 66075-110, Pará, Brazil

**Keywords:** pracaxi, sustainability, vegetable oils, amazon species, bioeconomy

## Abstract

Pracaxi (*Penthaclethra macroloba* (Willd.) Kuntze) is an Amazonian plant, traditionally used by the native population to treat health disorders such as inflammation, erysipelas, wound healing, muscle pain, ear pain, diarrhea, snake and insect bites as well as for cancer treatment. Other common uses include using the oil for frying, skin and hair beautification, and as an alternative source of energy. This review is focused on highlighting its taxonomy, occurrence and botanical origins, popular uses, pharmacology and biological activities, cytotoxicity, biofuel activity and phytochemistry in order to explore future therapeutic use and other applications. Pracaxi contains triterpene saponins, sterols, tannins, oleanolic acid, unsaturated fatty acids and long-chain fatty acids, with a high behenic acid value, which may serve for incorporation into drug delivery systems as well for the development of new drugs. These components are correlated with its anti-inflammatory, antimicrobial, healing, anti-hemolytic, anti-hemorrhagic, antiophidic, and larvicidal activities against *Aedes aegypti* and *Helicorverpa zea*, which ratify the popular/traditional uses. The species is nitrogen fixing; it is easy to propagate in floodplains and the terra firma, and it can be used for the reforestation of degraded areas. Additionally, the oil extracted from the seeds can leverage the bioeconomy of the region based on sustainable exploration.

## 1. Introduction

The Amazon is the largest tropical forest in the world with a large collection of medicinal plants used by the local population throughout generations as a therapeutic agent for maintaining health and as a cure for various illnesses [1]. Among the most popular species is *Pentaclethra macroloba* (Willd.) Kuntze, commonly known as pracaxi, pracachy, paracaxi (Brazil), gavilan (Costa Rica, Panama, Nicaragua), koloballi (French Guyana) [2,3]. Indigenous to the Amazon (Brazil, Guyana, part of Central and South America), the tree can measure an average of 14 m in height, growing in floodplains and terra firma areas; it is hyperdominant in the Amazon estuary [4,5,6,7].

**Figure 1:**
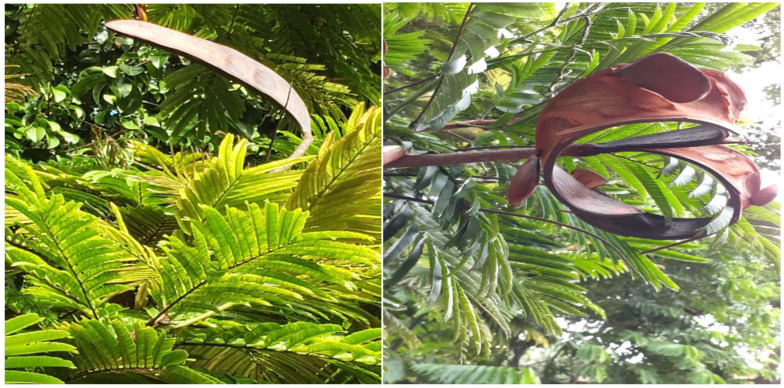
Pracaxi fruit and its seed (Ilha do Marajo, PA. By Luiz Morais).

*Pentaclethra macroloba* (Willd.) Kuntze produces fruits from which the seed oil is extracted (Figure 1); extracts, and powders are prepared from the bark, stems and branches, which are used by the population as a therapeutic agent for the treatment of muscle pain, inflammation, erysipelas and healing of ulcers, wounds on skin and snake bites [2,3,8,9,10,11,12,13,14,15,16]. The trunk bark has tannins and the tea is used to treat dysentery and diarrhea [5,10]. The pracaxi oil is popularly used as a hair treatment, for styling, increasing shine, and hair loss prevention. It is used in the treatment of stretch marks in adolescents and pregnant women, and when applied to skin spots, it combats hyperpigmentation [12,17]. In addition to its applicability for medicinal and cosmetic purposes, pracaxi oil is also used for cooking by some populations in the Amazon region of Brazil [3,14,18,19]. The seed husks are also used as cattle feed that is sought after due to its aroma and high protein content [20].

Studies found in the literature show evidence regarding its therapeutic activity in wound healing and burns [21,22,23], antimicrobial activity [10,24,25], non-cytotoxicity, genotoxicity and expression in eukaryotic cells [26,27], atherogenicity and thrombogenicity index [28,29]. Results also show its effect as an insecticide against larvae of the *Aedes aegypti* mosquito, vector of dengue and yellow fever, and *Helicorverpa zea* [30,31,32]. Antihemorrhagic, antinucleolytic and antiophidic activities were reported by Silva et al. [8], Silva et al. [33] and Carvalho et al. [34]. Robust scientific publications were also found regarding the characterization of the Pracaxi oil by gas chromatography (GC) techniques, thermal analysis (TG/DTG and DSC and DTA) and spectroscopy in the infrared region by Fourier transform (FTIR), oxidative stability—Rancimat [3,15,29,35], as well as pharmacological, phytochemical and toxicological assays [8,30,36,37,38,39,40].

Other evidence regarding the application of pracaxi extracts and oils are described in the literature, one such is the successful production of a pharmaceutical base with pracaxi oil for the treatment of burns and other skin conditions [22]. Pracaxi seed oil has also been used as the oil phase of controlled-release nanostructured drug delivery systems [41,42]. Pracaxi oil is involved in the development of a Pickering emulsion containing tocopherol, which provided the oil retention in the skin when applied topically (said emulsion received a patent granted by the National Institute of Industrial Property, No. BR 102015031604-6).

Pracaxi oil has been used in the cosmetic industry, as it is rich in fatty acids, has a high percentage of unsaturated fatty acids and a high content of behenic acid, which is a natural silicone [43,44]. Pracaxi oil has moisturizing, emollient, lubricating and softening properties, making it a useful addition to skin and hair products. These applications are supported by the numerous pracaxi oil hair products found on the market that boast anti-frizz, moisturizing, shine-promoting properties, and scar- and spot-lightening properties for skin care products [3,45,46,47]. Thus, the purpose of this work was to gather studies available in the literature related to the species *Pentaclethra macroloba* (Willd.) Kuntze that describe the popular use, occurrence, botany, phytochemical characterization, cytotoxicity, pharmacology, biological, nutritional, cosmetic activities and other applications for use in future research.

## 2. Taxonomy, Occurrence and Botany

*Pentaclethra macroloba* (Willd.) Kuntze is taxonomically categorized as follows: kingdom—Plantae; phylum—Magnoliophyta; class—Magnolipliopsida; order—Fabales; family—*Fabaceae*; genus—Pentaclethra; species—*Pentaclethra macroloba* (Willd.) Kuntze; synonyms: *Pentaclethra filamentosa* Benth and *Pentaclethra brevipila* Benth [5,7,48,49,50]. Found in northern Brazil, it typically grows on the banks of rivers, in lowland areas [4,7,12,14,51] and in some areas of dry land, mainly in the states of Amapá, Pará, Amazonas, Roraima and Acre [9,16,48,52,53].

*P. macroloba* is native to the Amazon and other South American countries (Guyana, Suriname, French Guiana, Venezuela, Colombia) and Central America and the Caribbean (Honduras, Jamaica, Nicaragua, Panama, Cuba and in Trinidad Tobago) [3,13,39,54]. It is a pioneer species that has a high density in the Amazon River estuary region [5,6,7,55,56].

**Figure 2:**
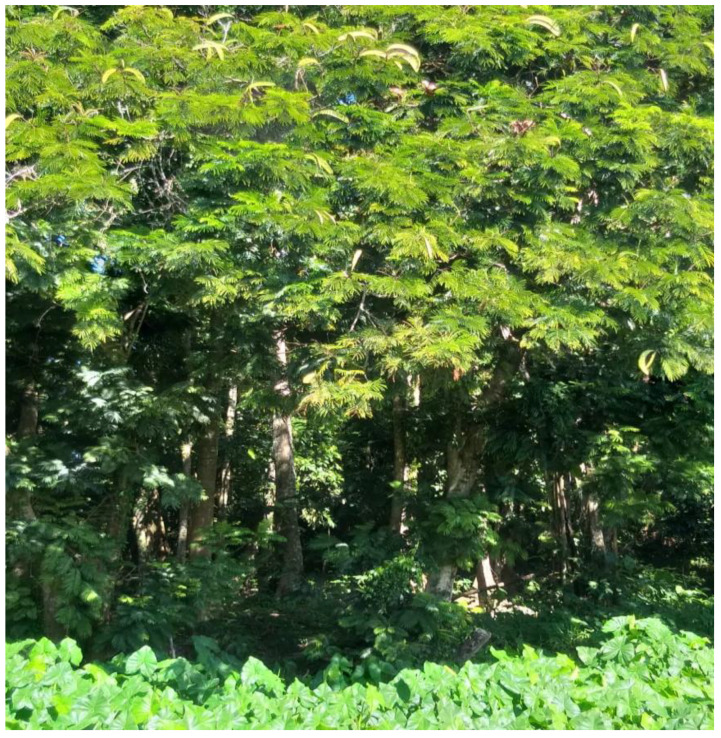
Pracaxi tree in the northern region of Pará planted at the Federal University of Pará.

The Pracaxi tree has an average height of 14 m (it can reach 37–45 m), the trunk is either straight or twisted, the leaves are bipinnate, and the inflorescence is in the form of terminal or subterminal spikes with white flowers (Figure 2) [4,39,57]. The name *Pentaclethra* comes from the Greek (penta) due to the imbricate structure of the five sepals and five petals joined at the base, which characterize the species of the genus [58]. It bears pod-shaped fruits measuring 16 cm to 45 cm, green or dark brown when ripe. This fruit is dehiscent, which means it opens abruptly and forcefully and projects the seeds at a great distance [56]. It flowers from July to September, and harvest takes place from January to June. Each fruit can contain four to eight seeds [5,7,12,48,59] with the average ripening time of fruits and seeds varies from 5 months (±3 months) to 3 months (±2 months), respectively [7]. From thirty-five pods on average, one kilo of seeds can be obtained, which contain approximately 30% oil on a dry basis [12].

Cruz and Barros [48] studied the biometry of pracaxi seeds and the results showed that the average mass was 5.42 g (approximately 100 seeds), the length was 42.2 mm, the width was 29.3 mm and the thickness was 8.3 mm. Soares et al. [59], when analyzing the anatomy of the fruits, seeds and seedlings of *P. macroloba*, described the fruits as being from 16 to 45.2 cm in length, from 4.1 to 7.3 cm in width, from 1.2 to 1.5 cm thick with 3 to 6 seeds per fruit. The seeds contain a pleurogram and are exalbuminous, glabrous, slightly wrinkled, shiny, eurispermic, with an ovoid, deltoid or elliptical shape, with a rounded apex and obtuse to acute base, measuring from approximately 3.0 to 6.1 cm in length, with width from 2.5 to 5.7 cm and thickness from 0.9 to 1.4 cm. The authors determined it to have an average fresh mass from 4.7 to 20 g, in contrast to other authors who described smaller masses [59]. In the study by Teixeira et al. [3], the average seed weight was 6.36 g, width was 31.19 ± 3.01 mm, length was 40.25 ± 9.60 mm and thickness was 9.60 ± 1.44 mm.

The oil cells are contained in the parenchyma cells of the cotyledons of the seeds. There is an air pocket between the cotyledons that allows it to float in the water, which favors seed dispersal [7]. Seed germination was identified to be hypogeal, the seedlings with bipinnate leaves, waxy cuticle and simple trichomes on the margins of the adaxial face and paracytic stomata on the abaxial face and the epicotyl in a slightly winding way [59]. During germination, the primary root emerges, the cotyledons open at a right angle, and the epicotyl appears, which later elongates, and the first pair of leaves appears. This seed germination occurs between 10 and 28 days and its development in the floodplain is fast [7,12,35,59]. The germination rate is 78 to 90% [7,48].

Studies by Eaton et al. [18], Shebistz and Eaton [60] pointed out that *Pentaclethra macroloba* (Willd.) Kuntze is critical for the recovery of soil nutrients in secondary forests, as it is the main nitrogen-fixing species [4], has great potential in forest regeneration and recovery of degraded areas. The *Fabaceae* family (to which pracaxi belongs) has a natural symbiotic relationship with nitrogen-fixing root microorganisms [61]. Pracaxi wood has little commercial value, although studies by Melo et al. [49] reveal its use by riverside populations as firewood, charcoal, and flooring, among other uses. Other research showed that the pracaxi tree has the potential to produce cellulose for papermaking [5,9,48,51,57]; however, its greatest potential lies in the seeds from which the oil, with all its medicinal properties, is extracted [5,7,10,12,14,21,25,33,54].

A study was carried out with *P. macroloba* in the State of Amapá, located in the extreme north of Brazil, to verify the spatial distribution pattern of the tree population in order to assist in the management and conservation strategies for this species. Determining the spatial pattern can identify more productive matrices and thus establish criteria for crops, which will enable sustainable exploitation of this species [5].

In regard to Dantas et al. [6], their studies provide foundational knowledge for the formulation of public policies for the sustainable management and conservation of natural resources of *P. macroloba* and its multiple uses. Thus, the importance of sustainable management of floodplain forests lies in allowing the diversity of their functions, their natural resources and ecosystem services, providing the opportunity for sustainable development of the human population in the Amazon.

## 3. Popular Use

The tree bark tea of the pracaxi plant is used to combat dysentery and acts as a strong vomitive to inhibit the effect of snakebite venom [3,5,9,14,48]. The tree is also used in cases of fever, skin rashes, lung and respiratory disorders, asthma, inflammation and bronchitis [48,50,62]. The leaves are also used to treat fungal infections [5,60,62]. The Warao meso-Indian people of the Orinoco Delta in eastern Venezuela use the tree bark and leaves to treat an array of endemic diseases such as diarrhea, bloody dysentery, helminths and external lesions. *P. macroloba* is documented in Warao pharmacopoeia [63].

The bark powder and seed oil are used in the treatment of skin ulcers, bedsores, erysipelas and wounds, inflammation, as well as muscle and joint pain [3,14,39,54]. The macerated bark can be applied as a poultice to combat the effects of snakebites [8]. An ethnobotanical and ethnopharmacological study carried out by Sarquis et al. [16] in a community of Rio Mazagão, in the State of Amapá in Brazil, reported that the leaves, inflorescence and bark are used for the treatment of inflammation and worms.

On the island of Combú-PARÁ in northern Brazil, Crespi and Guerra [14] interviewed residents who live on the subsistence of pracaxi seed collection and oil production. They stated that this population uses the oil for pain and wounds and various inflammations; they claimed a case of curing cancer, snake bite treatment (ingesting a tablespoon of pracaxi oil shortly after the bite), osteoarthritis and rheumatism treatment. The riverine people say that among the medicinal oils used on the island, “pracaxi oil has the greatest healing power”. Pracaxi oil is widely used by the population of the Amazon as a cosmetic hair care styler, shine promoter and for hair loss prevention; it is also used in the treatment of stretch marks in adolescents and pregnant women. When applied to skin blemishes, this oil reduces hyperpigmentation and discoloration [3,12,54].

## 4. Pharmacology and Biological Activity

The use of medicinal plants as a therapeutic treatment is pointed out by the World Health Organization (WHO) as a necessary resource to be valued since 1978 [64]. In vitro and in vivo studies have shown that the *P. macroloba* species has healing, anti-inflammatory, larvicidal, insecticidal, anti-hemorrhagic, anti-ophidian and anti-microbial activities related to the chemical constituents present in the plant [64,65].

### 4.1. Healing and Anti-Inflammatory Activity

Pracaxi oil has a predominance of unsaturated and long-chain fatty acids such as oleic acid and linoleic acid, and among vegetable oils, it has the highest concentration of behenic acid. Long-chain acids play an important role as precursors of prostaglandins, a potent vasodilator present in the inflammatory phase of wound healing [66]. Unsaturated fatty acids such as oleic acid reduce platelet aggregation with benefits in preventing coronary heart disease [67,68]. They are relevant to the intercellular lipid complex, and they act in maintaining the integrity of the skin barrier [23,69].

Pracaxi oil was used as a component of a topical formulation based on anhydrous silicone, with the objective of evaluating the action of fatty acids found in pracaxi oil for wounds, burns, surgical and traumatic scars demonstrated in a series of cases [18]. The study was evaluated for wound size, color and general appearance before and after treatment, supervised and photographed by an independent evaluator. It included a self-assessment questionnaire that measured the degree of patient satisfaction obtained with the product [22]. The results demonstrated the maximum degree of patient satisfaction on a scale of 1–10. This study was based on United States of America regulations, according to the Internal Committee for Experimentation on Humans and the Declaration of Helsinki. It was concluded that the pracaxi oil present in the formulation contributed to wound healing and improvement in the appearance of scars. The silicone base with pracaxi oil was named Pracasil^®^Plus marketed by the Professional Compounding Centers of America-PCCA [22]. Another case study conducted by Simmons et al. [21], in a 61-year-old male patient, with type 2 diabetes, who had an ulcer on the inner part of the leg and was treated with Pracasil^®^Plus, after using Mupirocin for sixty years. Complete wound closure was observed in three days.

### 4.2. Larvicidal and Insecticidal Activity

The use of pesticides is a common way to control pests, to protect agricultural crops and to prevent the transmission of zoonoses to humans. However, the use of synthetic pesticides has shown damage to the environment, as they are not selective, making pests more resistant and affecting the lives of other untargeted organisms in the ecosystem. The search for natural methods and products that minimize harmful effects is an alternative.

Larvicidal activity against *Helicoverpa zea* was identified in extracts prepared from *P. macroloba* seeds (extracted at 4 °C in 0.1 M phosphate buffer pH 7.0, containing polyvinylpyrrolidone) [30]. The authors reported that water-soluble active factors present in these extracts with molecular weight of approximately ≥3500 kDa, thermolabile and of a protein nature, would be responsible for the larvicidal effect, since these plant factors are known to inhibit the growth of larvae by being inhibitors of digestive enzymes [30]. The extract retarded the growth of larvae, reduced cell viability in the ovarian cell line of *Helicoverpa zea* and inhibited two of the three enzymes, trypsin and chymotrypsin, that are found in the larval midgut lumen, in a concentration-dependent manner of the extract [30].

Rathburn H. et al. [70] purified the crude aqueous extract of *P. macroloba* seeds and identified active compounds with molecular weights in the ranges between 38 and 35 and 6 and 9 kDa; within each range, trypsin inhibitors were detected. In the lower weight range, the substances exhibited stability after being heated at 100 °C for 30 min. The authors applied for patents for the invention of the method for obtaining the crude aqueous extract of *P. macroloba* seeds and its purification, as well as the trypsin inhibitor and other active components whose molecular weights are in the range of 38–45 and 6–9 kDa, and the protein weighing 43 kDa having the sequence Glu-Val-Val-Phe-Asp-Phe-Lys-Gly-Asp-Met-Met-Arg-Asn-Gly-Gly-His-Tyr-Tyr-Phe-Phe-Pro-Ala-Ala-Pro-Tyr-Gly-Gly-Gly-Asn-Leu-Leu-Ala Ala-Ala-Val (abbreviated nomenclature). The invention further relates to a process of protecting plants against the attack of insects, *Helicoverpa zea,* corn rootworms and similar insects subjected to these trypsin inhibitors which are contained in the range 38–45 and 6–9 kDa.

**Figure 3:**
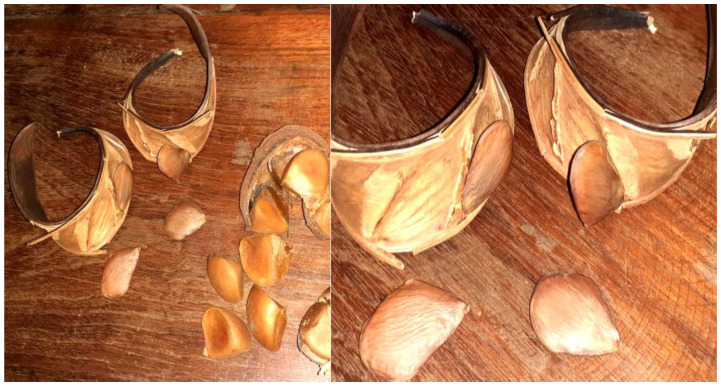
Pracaxi seed harvested from a farm on the island of Marajó, Para in Brazil (By Luiz Morais).

The *P. macroloba* extract also showed larvicidal activity against *Aedes aegypti* larvae. This activity was attributed to the four saponins present in the ethanolic extract of the seeds (Figure 3), bark of branches and wood that have the monodesmosid form with two to four sugars, the non-acidic portion being hederagenin and oleanolic acid and another carboxyl group at C-28 free isolated by Viana et al. [38]. The authors suggested that the carboxyl group present constitutes an essential unit for larvicidal activity. Saponin with two sugars in the acidic unit showed higher larvicidal activity with lethal concentrations (IC50 = 18.6 µg/mL ± 0.29) and it was determined that the lower the number of sugar molecules, the greater the larvicidal activity. Saponins that showed IC50 values lower than 100 µg/mL are promising larvicidal agents [31].

Santos et al. [32] carried out studies with oils from Amazonian plants, including pracaxi, to investigate the insecticidal potential, with a view to controlling the fall armyworm (*Spodoptera frugiperda*); the larvae of this butterfly species cause great economic losses as they attack farms extensively. Pracaxi oil showed efficacy on *Spodoptera frugiperda* eggs in this investigation.

### 4.3. Antiophidic, Antiproteolytic and Antihemorrhagic Activity

Plants are used by traditional populations in Brazil to curb the effects produced by snakebites. Pharmacological studies have shown that bioactive substances isolated from plant extracts produced antivenom effects in in vitro and in vivo tests, confirming the popular use of these species [34,67]. Aqueous extract of pracaxi showed anti-hemorrhagic, anti-nucleolytic and anti-ophidic properties. In vivo testing showed that this extract totally inhibited a metalloprotease (BjussuMP-I) present in the venom of *Bothrops jararacussu* snakes [8]. Subsequently, two triterpene saponins called macrolobin-A and B from *P. macroloba* found in the aqueous extract of pracaxi stem bark were purified and isolated [33]. These substances were able to neutralize the proteolytic, fibrinolytic and hemorrhagic activities induced by class P-I and P-III metalloproteases isolated from *B. neuwiedi* and *B. jararacussu* venom [33,59]. The authors concluded that the substances isolated from *P. macroloba*, macrolobin-A and -B, are important bioactive substances that could be associated with antivenom therapy for snakebites, serve as molecular models or constitute new therapeutic agents in the treatment of other diseases.

### 4.4. Antimicrobial Activity

In the literature, plants with antimicrobial activity due to the presence of tannins and other phenolic compounds were detected [24,68]. In studies carried out by Leal et al. [10], the ethanolic extract of *P. macroloba* stem bark was fractionated and the ethyl acetate and butanol fractions were evaluated against reference and clinical strains resistant to methicillin. The active fraction of ethyl acetate showed activity against Gram-positive (*Staphylococcus* spp. and *Enterococcus* spp.) and Gram-negative (*Pseudomonas aeruginosa*, *Acinetobacter* spp. and *Klebsiella pneumoniae*) multidrug-resistant bacteria. The ethyl acetate fraction presented ellagic acid as the main constituent, indicating that the presence of hydrolysable tannins would be responsible for the mechanisms of bactericidal action [10].

In another experiment, the agar diffusion test was used by Oliveira et al. [25] to test aqueous extracts of leaves, barks and fruits of *P. macroloba* at a concentration of 2.275 mg/mL against *Escherichia coli, Salmonella enterica serovar Enteritidis*, *Salmonella enterica serovar Typhimurium*, *Pseudomonas aeruginosa, Staphylococcus aureus*, *Enterococcus faecalis, Acinetobacter baumannii, Klebsiella ozaenae* and *Candida albicans* (inoculum at a concentration of 1 × 10^−3^ CFU/mL ATCC strains) compared to ciprofloxacin as a positive control at a concentration of 16.6 µg/mL. The extracts showed bactericidal activity against *Klebsiella ozaenae* and *Acinetobacter baumannii*.

Guimarães et al. [71] evaluated the in vitro antimicrobial activity of *P. macroloba* oil at concentrations from 0.156 mg/mL to 20 mg/mL by determining the minimum inhibitory concentration (MIC) and minimum bactericidal concentration (MBC) against the strain from *Staphylococcus aureus* (ATCC 25923). They identified that the oil did not show antibacterial activity in vitro against the *S. aureus* strain tested. In MIC, regardless of concentration, it resulted in microbial growth, and in CBM, it indicated that even at higher concentrations the oil did not inhibit microbial growth. However, the authors suggest that further studies should be carried out to test possible antibacterial properties of Pracaxi oil against Gram-positive bacteria, including *S. aureus*.

Rodrigues et al. [11] determined the antimicrobial activity of pracaxi oil and gelatin and chitosan emulsions containing 0.25% pracaxi oil against the *Staphylococcus aureus* strain (ATCC 25923). The chitosan/gelatin emulsion showed antimicrobial activity (MIC = 31.2 µg/mL), and the chitosan/gelatin/pracaxi oil emulsion had the same MIC value (31.2 µg/mL), so it became evident that the pracaxi oil did not contribute to the effective activity. When only pracaxi oil was analyzed, it did not reveal any antimicrobial activity against the evaluated microorganism. The data obtained corroborated the results of Guimarães et al. [71].

## 5. Cytotoxicity and Genotoxicity

Therapeutic agents of natural origin, even with proof of their benefits, must be submitted for genotoxicity and cytotoxicity evaluation to guarantee the safety of their application, as recommended by regulatory bodies such as ANVISA (National Health Surveillance Agency-Brazil) and FDA (Food and Drug Administration—USA), among others [72]. In studies by Leal et al. [10], the ethyl acetate sub-fractions of pracaxi stem bark did not show toxicity to eukaryotic cells.

Maistro et al. [26] performed tests to identify the cytotoxic and genotoxic potential of *Euterpe oleracea Martins* and *P. macroloba* oils. They employed two methods, the Comet assay in human lymphocytes in vitro and the MTT (3-4,5-dimethyl-thiazol-2-yl-2,5-diphenyltetrazolium bromide) which is based on the measurement of mitochondrial activity and reduces the MTT salt in Formazan. Concentrations of 2.5, 5.0 and 10 µg/mL of each oil and under the experimental conditions did not induce cytotoxicity and DNA damage.

In order to evaluate the safety of pracaxi oil, Pires et al. [40] investigated its in vitro effects against the human cell line HepG2/C3A (human hepatocarcinoma cells) regarding cytotoxicity, genotoxicity and gene expression. Cell viability assay by MTT method, Comet, Micronucleus and Reverse Transcriptase tests followed by polymerase chain reaction (RT-PCR) was used. The results revealed that the oil at concentrations of 31, 125 and 500 μg/mL did not reduce cell viability, indicating non-cytotoxicity. The results also revealed that the concentrations did not present a genotoxic effect and did not induce cell apoptosis. At a concentration of 500 μg/mL, pracaxi oil was able to stimulate the increase in mRNA of genes involved in mTOR (protein serine–threonine kinase) related to cell proliferation as well as the genes responsible for the metabolism of xenobiotics, cytochrome P 450 (CYP3A4, CYP1A2, CYP1A1) and those that refer to oxidative stress, glutathione peroxidase (GPX1). Finally, the authors concluded in their findings that the oil extracted from the seeds of *P. macroloba* at the concentrations analyzed was considered safe for use in humans.

## 6. Cosmetic Scar Lightening and Softening

Tyrosinase is the enzyme responsible for the synthesis of melanin from the amino acid L-tyrosine that occurs in the epidermis within melanocytes [73]. The main function of melanin is to protect the skin from solar radiation. However, the exaggerated production of this protein can lead to hyperpigmentation disorders such as melasma, age spots, freckles and sequelae of burns and other scars [47,73,74]. Vegetable oils, because they have hydrophobic components and phytoconstituents, can act as tyrosinase inhibitors by competing in the active sites of the enzyme by chelating copper ions. The development of products to minimize these effects, categorized as bleaching agents, is a major trend in the cosmetic industry that uses the bioactive compounds in some plants for this purpose [3,73,75].

Through a qualitative and quantitative assay, Teixeira et al. [75] evaluated in vitro the ability of seed oils from Amazonian plants, including pracaxi, to inhibit the mushroom tyrosinase enzyme, aiming at the incorporation of these oils into cosmetic products. They compared the performance using kojic acid as a positive control, and the enzyme L-tyrosine (Sigma-Aldrich, USA) was used as a standard. At the end of the experiment, pracaxi oil showed significant (*p* < 0.05) tyrosinase inhibition activity in relation to kojic acid.

Other studies have also identified hyperpigmentation reduction activity. Banov et al. [22] demonstrated in a case study that pracaxi oil, incorporated into a base containing anhydrous silicone, promoted lightening of surgical scars and burns.

Pracaxi oil has been used in the cosmetic industry in hair and skin care products due to its rich fatty acid content and high behenic acid content, with behenic acid being dubbed as the “natural silicone” [76]. Cationic surfactants whose main ingredient is pracaxi oil show clinically proven effectiveness in strengthening the hair fiber, improving manageability and increasing shine [77].

When it comes to cosmetic actives, Amazon Oil, a company based in Pará/Amazon-Brazil that supplies raw materials, works with riverside communities to ensure the seeds are collected in a sustainable way, has developed products containing pracaxi oil for use on the skin and hair. Products such as Nocaptone DMT LP^®^, BTM US30 LP^®^ and Angektase 40 PP^®^ were created for the treatment of skin conditions such as cellulite and stretch marks. The active ingredients for hair care promote anti-frizz properties, hair fiber structuring, emollient and chemical damage protection, formulated in the Behenshot 20DC LP^®^, Nutribalm 40 CD^®^, CM40CT^®^ and Chemshield 30 DQ LP^®^ [78]. Other popular cosmetic companies have launched products with pracaxi oil, including L’Oreal with the Kerastase^®^ hair line; Carol’s Daughter Pracaxi Nectar hair line “Styling by Nature” [77].

## 7. Nanostructures with Pracaxi Oil

Nanotechnology has allowed the production of very small structures (10^−9^ of a meter) capable of delivering drugs or other active substances for application in the pharmaceutical and cosmetic industries in order to maximize effects, minimize toxicity, and promote delivery at specific sites of action, among other benefits [79,80,81]. Mattiazzi at al. [42] obtained nanocapsules with the polymer PCL (Poly(ε-caprolactone) whose core was formed by pracaxi oil and umbiquinone and developed and validated the RP-HPLC-UV Assay Method for the nanocapsules.

Solid particles were used as stabilizers to replace classic surfactants to obtain a Pickering emulsion based on pracaxi oil and containing tocopheryl acetate for topical use. The emulsion droplets had a size of 677.2 nm ± 0.2 and were stable according to a stability assay conducted. In an in vitro skin permeation test, using pig ear, it showed retention in the dermis and epidermis, favoring the action of pracaxi oil and tocopherol for the proposed purpose. Work carried out by this research group gave rise to the Patent Letter entitled “Pickering Emulsion with Pracaxi Oil (*Pentaclethra macroloba*) containing vitamin E (tocopheryl acetate) for topical use” Letter Patent Nº BR 102015031604-6 granted to the Federal University of Pará by National Institute of Industrial Property—INPI issued in 2020 (Brazil Ministry of Economy—INPI 2020).

## 8. Biofuel with Pracaxi Oil

Vegetable oils and fats have been studied as alternative energy sources. In this context, there has been an increased interest in plants from the Amazon aiming at the production of biodiesel [82,83,84]. Lima et al. [35] synthesized biodiesel from pracaxi oil. The product’s high thermal stability and other physico-chemical properties met the specifications contained in Resolution 45/14 of the National Petroleum Agency (RANP 45/14), ASTM D6751 (American Society for Testing and Materials) and European Committee Standard EN 14214. According to the authors, it is a candidate for inclusion in the Brazilian Energy System.

## 9. Phytochemistry—Substances Identified in *P. macroloba* Species and Their Applications

Substances of pharmacological interest were identified and isolated from the seed oil, the bark (trunk) and wood (twig) extracts of the *P. macroloba* species, whose activities were evidenced by several authors [17,44,85]. Studies carried out by researchers from the Brazilian Agricultural Research Corporation (EMBRAPA) identified sterols in pracaxi oil, with stigmasterol being the major component (53.96%), followed by ß-sitosterol (33.96%) and campesterol (6.28%) [36]. Proteins were also identified in extracts of pracaxi called PmSTI and PmLTI (“*P. macroloba* small trypsin inhibitor and *P. macroloba* large trypsin inhibitor”) with insecticidal and larvicidal activity [70]. Wilbert and Haiek [63] performed screening on the *P. macroloba* species used by the Warao society and identified sterol saponins, tannins, flavonoids, and polyphenols compounds. These findings were important to insert in the Warao pharmacopeia.

Other structures of pharmacological interest were isolated and identified from the *P. macroloba* species. Viana et al. [37] and Viana et al. [38] isolated and identified four triterpene saponins of the monodesmosidic type containing genins such as hederagenin and oleanolic acid from the ethanolic extract of the stem barks: 3ß-O-[ß-D- glucopyranosyl-(1→4), α-L-rhamnopyranosyl-(1→2)]-α-L-arabinopyranosyloleanolic acid (1); 3ß-O-{[ß-D-glucopyranosyl-(1→3)-α-L-rhamnopyranosyl-(1→2)],ß- D-g l u c o p y r a n o s y l-(1→4)}-α-L-a r a b i n o-pyranosyloleanolic acid (2); 3ß-O-{[ß-D-glucopyranosyl-(1→3)-α-L-rhamnopyranosyl-(1→2)],ß-D-glucopyranosyl-(1→4)}-α-L-arabinopyranosylhederagenin (3); 3ß-O- {[ß-D-glucopyranosyl-(1→4)-ß-D-glucopyranosyl-(1→3)-α-L-rhamnopyranosyl-(1→2)], ß-D-glucopyranosyl(1) →4)}-α-L-arabinopyranosyloleanolic acid (4). These showed larvicidal activity for *Aedes aegypti* [37,38]. Later, Silva et al. [33] also identified two of these triterpene saponins in the aqueous extract of the stem bark of *P. macroloba*, which they called Macrolobin A and B capable of inhibiting metalloprotease from snake venom of the genus *Botrops*, with antiproteolytic and antinucleolytic activity anti-hemorrhagic [8,33,34].

Polyphenols that occur in plant metabolism are potent antioxidants with beneficial effects on human health [86]. The presence of phenolic compounds was revealed by Teixeira et al. [3], when they demonstrated high antioxidant activity of pracaxi oil (31.92 to 54.05%) found in mg/GAEkg of gallic acid. These values were higher when compared to corn (11.1%), grapes (13.4%), soybeans (17%), flax (19.3%), being similar to rice bran (23.7%) and sunflower (23.9%). Serra et al. [87] identified the presence of γ- tocoferol, δ- tocoferol, α-tocotrienol, β- tocotrienol, γ- tocotrienol (total vitamin E 597,36ppm).

Le Cointe [2] stated that pracaxi bark is an astringent and is rich in tannins; later, Leal et al. (2011) confirmed the presence of tannins with bactericidal activity in an ethyl acetate fraction of the ethanolic extract of *P. macroloba* bark and ellagic acid identified as the main constituent in this fraction. Ellagic acid is a polyphenol derived from gallic acid that has antioxidant, antiadipogenic activity and high potential for cancer prevention and treatment [88].

Long-chain fatty acids and unsaturated fatty acids are predominant in pracaxi oil; however, the high behenic acid content makes it unique, as it has a higher value than all vegetable oils (nine times more than peanuts) and animals fats already studied [3,15]. The high levels of mono- and polyunsaturated fatty acids in pracaxi oil indicate low levels of atherogenicity (0.02–0.23) and thrombogenicity (0.14–0.70), evidencing the benefits in the prevention of coronary diseases [28,29].

Studies by Costa et al. [15], Bezerra et al. [28]; Lima et al. [35], Pereira et al. [29], Serra et al. [87] and Teixeira et al. [3] characterized the oil by gas chromatography coupled to GC/MS mass spectrometry and revealed the presence of palmitic acid (1.43–1.95%) in pracaxi oil (1.43–1.95%), as well as stearic (2.68 -5.09%), oleic (47.30–53.55% majority), linoleic (11.70–13.05%), linolenic (0.13–1.23%), behenic (16.13–22.60%), lignoceric (10.44–12.49%), lauric (0.10–1.20%), myristic (0.09–0.71%), and arachidic (1.0–12.30%) acid. In addition, twenty-one triacylglycerols (TAGs) were identified by Teixeira et al. [3], ten indicated the presence of behenic acid BeBeLg; POBe; SOBe; SOLg; BeBeO; LgOBe; LgLgO; OOP; OOS; OOBe; OOLg; BeBeLi; LgLiBe; PLiO; OOO; OLiBe; OLiLg; OOLi; LiLiBe; LiLiLg and LiLiO (P-Palmitic, S-Stearic, O-Oleic, Li-Linoleic, Be-Behenic, and Lg-Lignoceric). Because pracaxi oil shows a unique profile, Funasaki et al. [89] and Teixeira et al. [3] suggested that behenic acid could be used as a chemical marker, which will facilitate the identification of adulteration.

## 10. Obtaining the Oil and Physicochemical Characterization

### 10.1. Methods of Oil Extraction and Refining

The traditional extraction of pracaxi oil carried out by communities in the Amazon is performed by cooking the seeds in an artisanal way, while the industrial process involves cold pressing using a hydraulic press. Other methodologies described in the literature use supercritical fluid [3]. In the supercritical fluid methodology, the authors reported the advantage of clean extraction, free of organic solvents, shorter processing time, and preservation of constituents, composing a green technology. Vegetable oils with a high content of free fatty acids can be de-acidified using this method. A liquid–liquid extraction technique for refining pracaxi oil using ethanol was also conducted by Pereira et al. [53].

### 10.2. Physicochemical Characterization of Pracaxi Oil

The thermal behavior of pracaxi oil was evaluated by thermogravimetric analysis (TG) and its derivative (DTG) showed that the stability of the oil was maintained until the temperature of 220 °C, from which the decomposition of fatty acids occurred, reaching the greater mass loss (98%) between 400 and 450 °C [15,35].

The results of the analysis by differential scanning calorimetry (DSC) between temperatures −80 and 60 °C showed a curve with two exothermic peaks indicating that the crystallization of TAGs was obtained [3,29]. The first peak around 5.55 °C relating to the crystallization of TAGs composed of saturated fatty acids corresponding to a fraction rich in behenic and lignoceric acids and the second close to −43.22 °C probably related to TAGs containing mono- and poly-unsaturated acids such as oleic and linoleic acid [3,29,90]. The transition phase for melting pracaxi oil demonstrated by Teixeira et al. [3] occurred between 11.90 and 14.68 °C indicated by an endothermic event referring to mainly di- and tri-saturated saturated fatty acids [3,29]. Pracaxi oil presents fat crystals under refrigeration between 4 and 10 °C, but it is completely liquid above 20 °C [3].

Costa et al. [15] analyzed the thermal behavior by differential thermal analysis (DTA) of three samples of pracaxi oil in the temperature range from 0 to 700 °C. They showed three endothermic peaks at 330, 440 and 540 °C relative to boiling and combustion of the samples. Between temperatures 40 and 450 °C, an exothermic peak occurred, which corresponds to the decomposition of C18 saturated and C 18:1 unsaturated fatty acids, C 18:2, and C18:3, probably due to oxidation of the raw material.

Fourier Transform Infrared spectroscopic analysis showed a carbonyl elongation band at 1746 cm^−1^ and C–O bond vibration at 1238, 1164 and 1100 cm^−1^ (triacylglycerol ester bond). Close to 3000 cm^−1^, high intensity bands were identified at 2930 and 2855 cm^−1^ corresponding to HC=, and –CH_3_ groups, respectively. A low-intensity band was also revealed at 3010 cm^−1^ referring to the elongation of unconjugated C–H double bonds, symmetrically disubstituted in the cis position. There is an out-of-plane CH deformation at 723 cm^−1^ and elongation of the cis structure (–CH=CH–) at 1654 cm^−1^. Moderate-intensity bands related to axial deformation of C–H aliphatic groups at 1464 and 1377 cm^−1^ are also presented [15].

As already described in this work, the GC/MS analysis of pracaxi oil detected oleic acid (majority), as well as behenic, linoleic, lignoceric, palmitic, arachidic, myristic, lauric, and linolenic acids, with a predominance (60–67%) of unsaturated (mono- and polyunsaturated) and long-chain fatty acids [3,15,28,29,35]. The oxidative stability of pracaxi oil was analyzed by the method standardized by the European Standard EN 14112, adopted in Brazil by the ANP (National Agency of Petroleum, Natural Gas and Biofuels) based on ANP nº 14/2012; the RANCIMAT equipment was used with the period of induction (IP) measured by conductivity. Of the three samples evaluated, the results were the following: sample 1 IP equal to 8.52 h, sample 2 IP equal to 10.32 3 h and sample 3 IP equal to 10.42 h, which demonstrated acceptable oxidative stability as it was in accordance with the limit adopted by the ANP that requires at least 6 h of testing [15].

## 11. Patents Related to Pracaxi Oil

According to Oliveira et al. (2019), there is a considerable number of published patents involving pracaxi oil until 2019, fifty-six patents granted at the time on the LENS base (LENS ORG) and twenty-six on the WIPO (Word Intellectual Property Organization) platform, with the following amongst them:

“*Pentaclethera macroloba* protein having insecticidal properties”, inventors Rathburn H. et al., patent USOO567268OA, granted in 1997 (first patent granted).

“Permeation Enhancers for Topical Formulations” inventors Daniel Banov, August S. Bassani, patent US8906397B2, granted in 2014.

“Mixture of Betamethasone and Tranilast with a Transidermal Gel for scar treatment”, inventor Daniel Banov, patent US9173940B1, granted in 2015.

“Topic Pharmaceutical Bases for the Treatment of Skin Diseases, inventor Daniel Banov, patent US 09775872B2, granted 2017.

“Pickering Emulsion with Pracaxi Oil (*Pentaclethra macroloba*) containing vitamin E (tocopheryl acetate) for topical use” Letter Patent Nº BR 102015031604-6 granted to the Federal University of Pará by National Institute of Industrial Property—INPI issued in 2020 (Brazil Ministry of Economy—INPI 2020).

## 12. Conclusions

The species *P. macroloba* has been widely studied and a relevant portion of scientific production supports the applications of its popular use. Although there are some gaps regarding the mechanism of action involved in the activities, it is worth mentioning that the use was considered safe at the doses tested for cytotoxicity and genotoxicity to humans. The inputs from the seeds can be used in the pharmaceutical, food, cosmetic, agricultural and biofuel industries. Research can be implemented using isolated chemical substances as a model for new drugs. There are well-established studies regarding species identification, management, seed production potential, and information that support the implementation of policies that respect the traditions of the peoples of the Amazon, the biome and sustainable extractivism in order to keep the forest standing and leverage the region’s bioeconomy.

## Data Availability

Not applicable.
